# Experts’ Perceived Patient Burden and Outcomes of Knee-ankle-foot-orthoses (Kafos) Vs. Microprocessor-stance-and-swing-phase-controlled-knee-ankle-foot Orthoses (Mp-sscos)

**DOI:** 10.33137/cpoj.v5i1.37795

**Published:** 2022-02-25

**Authors:** B. Brüggenjürgen, F. Braatz, B. Greitemann, H. Drewitz, A. Ruetz, M. Schäfer, W. Seifert, F. Steinfeldt, C. Weichold, D. Yao, C. Stukenborg-Colsman

**Affiliations:** 1 Institute for Health Services Research and Technical Orthopedics, Orthopedic Department - Medical School Hannover (MHH) at DIAKOVERE Annastift Hospital, Hannover, Germany.; 2 Klinik für Unfallchirurgie, Orthopädie und Plastische Chirurgie Universitätsmedizin Göttingen, Georg-August-Universität, Göttingen, Germany.; 3 RehaKlinikum Bad Rothenfelde, Klinik Münsterland, Bad Rothenfelde, Germany.; 4 Abteilung Orthetik, Otto Bock HealthCare Deutschland GmbH, Göttingen, Germany.; 5 Klinik für Konservative Orthopädie, Katholisches Klinikum Koblenz, Montabaur, Germany.; 6 Orthopädie-Technik, Pohlig GmbH, Traunstein, Germany.; 7 Technische Orthopädie, Seifert Technische Orthopädie GmbH, Bad Krozingen, Germany.; 8 Fachklinik und Gesundheitszentrum, Johannesbad Raupennest GmbH & Co. KG, Altenberg, Germany.; 9 Technische Orthopädie, Stiftung Orthopädische Universitätsklinikum, Heidelberg, Germany.; 10 Foot Department and Technical Orthopedics, Orthopedic Department - Medical School Hannover (MHH) at DIAKOVERE Annastift Hospital, Hannover, Germany.

**Keywords:** Knee Instability, Ankle Foot Orthoses, KAFO, Microprocessor Orthoses, MP-SCCOs, Poliomyelitis, Patient Burden, Quality of Life, Survey

## Abstract

**BACKGROUND:**

Patients with neuromuscular knee-instability assisted with orthotic devices experience problems including pain, falls, mobility issues and limited engagement in daily activities.

**OBJECTIVES:**

The aim of this study was to analyse current real-life burden, needs and orthotic device outcomes in patients in need for advanced orthotic knee-ankle-foot-orthoses (KAFOs).

**METHODOLOGY:**

An observer-based semi-structured telephone interview with orthotic care experts in Germany was applied. Interviews were transcribed and content-analysed. Quantitative questions were analysed descriptively.

**FINDINGS:**

Clinical experts from eight centres which delivered an average of 49.9 KAFOs per year and 13.3 microprocessor-stance-and-swing-phase-controlled-knee-ankle-foot orthoses (MP-SSCOs) since product availability participated. Reported underlying conditions comprised incomplete paraplegia (18%), peripheral nerve lesions (20%), poliomyelitis (41%), post-traumatic lesions (8%) and other disorders (13%). The leading observed patient burdens were “restriction of mobility” (n=6), followed by “emotional strain” (n=5) and “impaired gait pattern” (n=4). Corresponding results for potential patient benefits were seen in “improved quality-of-life” (n=8) as well as “improved gait pattern” (n=8) followed by “high reliability of the orthosis” (n=7). In total, experts reported falls occurring in 71.5% of patients at a combined annual frequency of 7.0 fall events per year when using KAFOs or stance control orthoses (SCOs). In contrast, falls were observed in only 7.2 % of MPSSCO users.

**CONCLUSION:**

Advanced orthotic technology might contribute to better quality of life of patients, improved gait pattern and perceived reliability of orthosis. In terms of safety a substantial decrease in frequency of falls was observed when comparing KAFO and MP-SSCO users.

## INTRODUCTION

Orthotic devices, in particular knee-ankle-foot-orthoses (KAFOs) are well accepted for treating knee instability in neuromuscular disease and central nervous system conditions. However, burden of disease and individual demands of patients have been rarely studied. Knee instability conditions can cause several problems, including pain, falls, range of mobility issues and limited engagement in daily activities, which could be alleviated with the use of better orthotic devices.^1,2^

A KAFO is usually prescribed when other types of bracing like ankle-foot-orthoses (AFO) cannot adequately control knee instability because of weakness (e.g., quadriceps weakness) or ligament laxity.^3^ Patients suffering from knee instability due to neuromuscular disease (e.g., after acute poliomyelitis, incomplete spinal cord injury, or femoral nerve lesions), benefit from KAFOs with locked or posterior off-set orthotic knee joints by preventing the paretic or paralyzed leg from collapsing and to aid in safe ambulation.^2,4^ However, a locked knee precludes knee flexion during swing and, thus, requires compensatory mechanisms to achieve sufficient toe clearance.^2^

Stance control orthoses (SCO) allow users to flex their knee during the swing phase to reduce the compensations of hip hiking and circumduction. However, these benefits are mostly limited to walking on level surfaces, because the difficulty to relock the orthotic knee joint on non-level terrains results in limitations in function and safety for walking on uneven terrain, stairs, ramps, or with varying speed.^4–6^ In particular, patients’ limited ability to walk and maintain conditioning results in a high risk of falling due to deconditioned coordination and balance.^1^

A microprocessor-swing-and-stance-controlled knee-ankle-foot-orthosis (MP-SSCO) provide both swing and stance phase control for patients suffering from paralysis or paresis of the muscles that stabilize the knee. C-Brace (Ottobock, Duderstadt, Germany) is the only MP-SSCO currently holding a market authorization in the US and the European Union. The microprocessor control enables dampening of knee flexion during weight-bearing and speed-adapted control of knee flexion and extension during the swing phase.^7^ This feature enhances patients’ confidence in knee joint function, increased walking speed and energy efficiency, and improved safety for walking on uneven terrain, stairs, and ramps.^8^ Limited published data could only be obtained for one other microprocessor-controlled orthosis type, a microprocessor-controlled-stance-controlled orthosis (MP-SCO).^9^

Independence for individuals with lower limb motor disabilities is a key issue in their daily routine and can be enhanced with the use of assistive devices to promote their participation in social activities and in living a self-sufficient life.^10^ Hence, the key issue in orthotic care is utilizing the appropriate orthosis (KAFO or MP-SSCO) to address users’ needs and expectations.^11–13^ Furthermore, real-life settings are difficult to scrutinize due to the multitude of underlying disease states as well as different settings and patients’ behaviors that complicates pragmatic real-life trials.

Aim of this study was to analyse the current real-life burden of patients in need for advanced orthotic KAFOs, their needs and patient relevant outcomes as well as the potential benefit of a MP-SSCO on patient outcomes and care processes.

## METHODOLOGY

### System Instrumentation

An observer-based semi-structured interview survey with experts in the field of KAFO/MP-SSCO fitting was used. Expert participants were selected based on a) meeting orthotic clinical and technical care expertise qualifications (both physicians and orthotists) and b) having been involved in orthotic care for more than 5 years both with KAFOs as well as MP-SSCO in a German orthotic care centre. Furthermore, participants were only selected, if willingness to devote time and interest to the specific topic was stated, as well as informed consent to participate was provided. As this survey was neither a notifiable clinical study according to §47 par. 3 MDPG nor an epidemiologic study with individual patient reference an ethics committee vote did not apply.

Semi-structured interviews are employed in qualitative interview research where the order and content of questions in an interview can be modified to deepen the exploration of a research topic according to the response of the interviewee.^14^ The interviews were conducted by phone and survey questions were comprised of the following areas: participants’ orthotic experience, challenges in orthotic rehabilitation, patient burden and severity of this burden, patient needs and benefits, patient relevant outcomes, rehabilitation under delivery of new orthotic devices, severity and frequency of falls, adverse events other than falls. Queries were phrased using MP-SSCO as the product group term with C-Brace (Otto Bock, Duderstadt, Germany) being presented to the interviewees as an example once or on request.

The information collected in the interviews was transcribed and was prepared for a content analysis. Content analysis is described as a method to classify written or oral materials into identified categories of similar meanings.^15^ The analytic process of the qualitative interview component approximated the following steps: determination of category and levels of abstraction, the development of inductive categories from material, the revision of categories, the final working through text, and the interpretation of results.^16^ Quantitative questions were reported with descriptive statistics.

## RESULTS

Eight clinical experts confirmed participation in the semi-structured telephone interview lasting approximately one hour in duration. Interviews were performed from 2020-11-09 to 2021-02-03. All participants (7 male, 1 female; 4 physicians and 4 orthotists) had long-term experience in prescribing, fitting, and delivering acute as well as subacute and chronic rehabilitation care both for KAFOs and MPSSCO users. On average, experts’ centres prescribed or delivered 49.9 KAFOs per year and 13.3 MP-SSCOs (C-Brace only) since product availability to patients. The treated population is comprised of patients with incomplete paraplegia (18%), peripheral nerve lesions (20%), polio-myelitis (41%), posttraumatic lesions (8%) and other disorders, including stroke sequelae (13%).

### Challenges in orthotic rehabilitation

Half of the experts (n=4) considered a correct indication and diagnosis as a key challenge for patient rehabilitation in orthotic care, and in particular for MP-SSCO: 38% emphasized the need for intensive patient support and guidance, especially for understanding and using the functionality of the orthosis. A safe stance phase, safe handling by the patient and a well-fitting orthosis with proper alignment of the components as well as the challenges in approaching reimbursement bodies were mentioned by two interviewees. Categories reported once only were: Standardized assessments for orthosis selection, risk of falling due to stiff knee, foot clearance, dressing/undressing in daily routine, early integration of orthosis into therapy and training, gap between technical feasibility and return to full participation in real life as well as a need towards an interdisciplinary approach.

Regarding structure and process of rehabilitation, 7 out of 8 of the interviewees observed a relevant change when switching to MP-SSCO. Changes were mentioned in particular with regard to a more intensified initial education phase in MP-SSCO patients (57% of those reporting change) as the understanding of the orthosis’ potential was considered essential (43%). Despite the intensity of early rehabilitation activities, a shorter overall process was reported (57%). Seven interviewees reported several extraordinary case reports, such as a female, middle-aged, still active teacher with a lower limb amputation on the contralateral side and a substantial paralysis on the supplied side being instantly able to return to independent ambulation. A further example provided was a young, female patient with a complete femoralis paresis after polytrauma suffering extremely from abrupt cessation of her active life who was able to return to independent ambulation with the committed support of the centre’s team. Categories of extraordinary cases could be categorized as unexpected regain of mobility and ambulation, return to ambulation without crutches, possibility of fitting an orthosis in difficult posttraumatic situations where orthotic care had been unavailable previously, and cases of fully unexpected clinical benefit and occupational rehabilitation.

### Patient Burden

**Figure 1:**
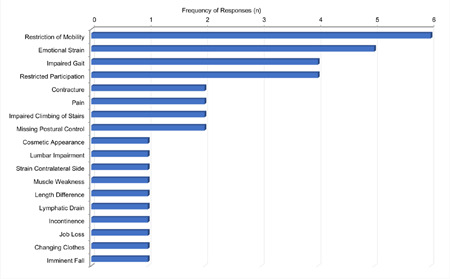
Patient impairments and burden as perceived by experts

In terms of expert-observed burden on patients due to impairment, “restriction of mobility” ranked highest among the queried three most serious items (n=6), followed by “emotional strain” (n=5) as these patients often suffer from severe progressive and/or continuously deteriorating conditions with multiple comorbidities. The third item was “impaired gait pattern” (n=4), including “lack of symmetry” and “negative impact on full social participation”, exemplified by one interviewee as issues with the aesthetic appearance (both n=4). Further impairments observed were “extension contractures or flexion restrictions”, “painful conditions”, “impaired climbing of stairs” and “lack of postural control”, as those patients may have impaired stability and/or not have the ability to stand (n=2). (**Figure 1**)

**Figure 2:**
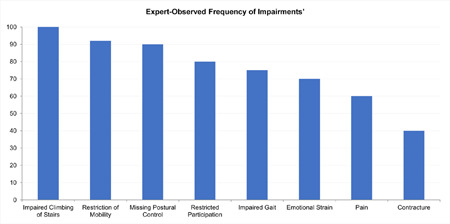
Expert-observed frequency of impairments (reported more than one time). Categories (always, often, sometimes, seldom, never) were transferred to 0-100-scale with 100 representing the highest frequency “always”.

For those serious impairment items reported at least by two experts, “impaired climbing of stairs” obtained the highest observed frequency followed by “restriction of mobility”, “lack of postural control”, “restricted participation”, impaired gait”, and “emotional strain”. “Pain” and “contracture” were reported sometimes or rarely with regard to frequency. (**Figure 2**)

### Potential Patient Benefits

**Figure 3:**
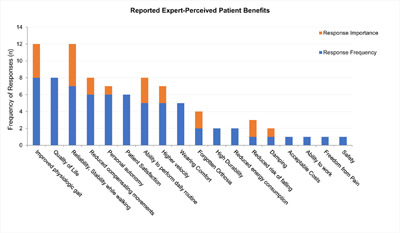
Reported expert-perceived patient benefits.

Experts judged “quality of life” of patients (n=8) as well as “improved gait pattern” (n=8), followed by “high reliability of the orthosis” (n=7) as the most relevant domains of potential patients’ benefits from optimal delivery of orthotic care. “Patient satisfaction”, “personal autonomy” and “reduction in compensatory mechanisms” were reported as relevant by six experts. The items “ability to perform daily routine”, “wearing comfort” and “higher velocity” obtained a frequency of five reports. (**Figure 3**)

When valuing each benefit’s importance to patients and selecting the three most relevant aspects, “perception of safety and high stability while walking” ranked highest (n=5), followed by “physiologic gait” (n=4) and the ability for “participation in daily life” (n=3). “Not having to think about the orthosis”, i.e., no need for conscious orthotic control, “prevention of falls”, “mobility” and “ability to swing” were seen by two experts amongst the three top-ranking domains of patient need. (**Figure 3**)

### Outcomes Assessment in Orthotic Device Alignment

**Figure 4:**
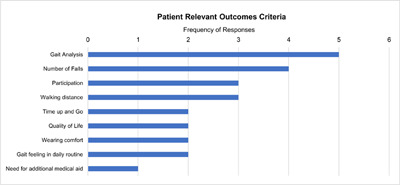
Relevance of suggested patient assessment criteria.

Furthermore, experts provided suggestions to implement specific tools for patients’ assessment in clinical routine covering both patient-relevant orthotic-device related outcomes as well as quality assurance aspects. In this context, “gait analysis”, e.g. via video, was reported most often with high relevance (n=5), followed by “number of falls in patient history” (n=4), “participation” and “walking distance” (n=3). (**Figure 4**)

### Orthosis-specific safety and long-term outcomes

#### Frequency of Falls and Resource Use - KAFO

When using KAFOs or stance control orthoses without microprocessor control (SCOs), falls were estimated to occur once weekly (standard deviation (SD) 0.0) in 4.0 % (SD=9.9) of patients. Monthly falls were seen in 23.8% (SD= 28.3) and annual falls in 43.8% (SD=30.0) of patients with a frequency of 1.25 (SD=0.9) and 3.19 (SD=1.5), respectively. In total, falls were reported to occur in 71.5% of patients at a combined annual frequency of 7.0 fall events per year when using KAFOs or SCOs.

Serious falls may result in the need for medical care. Healthcare resource use related to falls with non-microprocessor-controlled knees (non-MPKs) was estimated to require hospital care in 4.0% (SD=3.7) and non-hospital medical care in the out-patient setting in 19.3% of fall cases (SD=17.5). 76.7% (SD=20.1) of total falls were considered not to require any health professional care.

### Long-term Consequences

**Figure 5:**
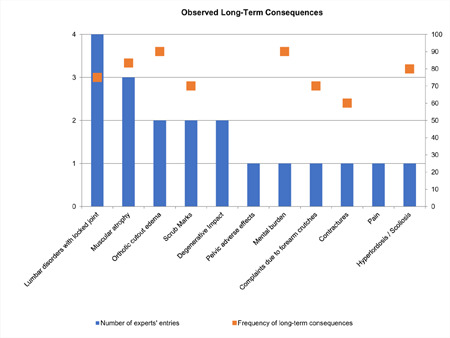
Observed long-term consequences (primary axis: number of experts reporting, secondary axis: Transposed frequency of consequences in percent of time occurring - Categories (always, often, sometimes, seldom, never) were transferred to 0-100-scale with 100 representing the highest frequency “always”.)

Wearing KAFOs and SCOs is associated with both adverse effects and long-term consequences. Adverse effects include “excessive lumbar loading with lack of trunk stability” (n=3), “impairment of gait” (n=2) and “stiff limb”, “rollator dependency”, “fitting problems”, “risk of luxation in hip replacement patients” and “noise” which were each reported only once. Regarding long-term consequences, most experts considered “lumbar disorders with a locked knee joint” (n=4) as the most relevant item, followed by “muscular atrophy” (n=3). “Orthosis cut-out oedema”, “scrub marks” and “degenerative impact” were reported being relevant by two experts. “Pelvic adverse effects”, “mental burden”, “physical discomfort due to forearm crutches”, “contractures”, “pain” and “hyperlordosis / scoliosis” were reported as relevant by one expert each. “Scrub marks” were reported both as adverse effect and long-term consequence. All long-term consequences were considered to occur “always” to “sometimes” in frequency (**Figure 5**).

#### Frequency of Falls and Experiences with MPK fitting

Experts termed orthotic care with an MP-SCCO as outstanding only when patients benefitted considerably in real life settings (following a sound trial fitting), despite ambiguous diagnostic results during the initial assessment (n=4). Further outstanding experts’ experiences were successful fitting of orthosis in complex post-traumatic and postoperative situations (n=3) and the regain of mobility in the patients´ daily routine (n=2).

The frequency of falls in MP-SCCO users was estimated on an annual basis. Falls were observed to occur in 7.2 % (SD=10.3) of all patients with an annual frequency of 2.2 (SD=3.0) fall events per year.

### DISCUSSION

Burden of patients in need of KAFOs was analysed with an experts’-based interview survey. Restriction of mobility, emotional strain and impaired gait were reported as major impairment aspects. Potential for improving care in patients who use non-microprocessor-controlled KAFOs is seen in particular with regard to quality of life (QoL), gait, and reliability of the orthosis. The experts underscored the importance of appropriate outcomes assessment criteria, such as gait analysis, orthosis safety (prevention of falls), and participation in activities of daily living. In terms of frequency of falls, a substantial difference between non-microprocessor-controlled KAFOs and MP-SSCOs was reported.

In our study improved gait pattern, QoL and high reliability of the orthosis were most often reported as important outcomes to patients. This is largely in line with health care professional (HCP) estimates reported by O’Connor et al., where comfort, confidence in mobility and increased stability were most often reported as being very or extremely important to patients.^1^ Though QoL in our study was most frequently stated as a relevant patient benefit of optimal orthotic care, no expert valued QoL or patient satisfaction amongst the three most important domains. This lack of perceived high importance might be due to the fact that QoL comprises different physical and psychological domains of health-related QoL and, hence, results in an improvement, if a majority of other aspects of benefit have been achieved. Adding to this line of reasoning is the fact that retraining of compensatory gait patterns, such as circumduction, requires time and, hence, may delay an early, straightforward improvement in QoL.^17^ Furthermore, the reported experts’ perception might be influenced by the German healthcare reimbursement decision making process, where QoL has just recently gained more importance.

Unlike Yang et al., we conducted interviews with experts caring for patients in need for lower limb orthoses.^10^ However, despite not directly involving patients’ responses, research of health-related QoL did show that the results obtained from attending HCPs considerably overlap with patients’ individual feedback, resulting in reasonable agreement that make the HCPs’ perception a valuable source of assessment.^18^ However, when assessing individual patients, there was some discordance between scores obtained from HCPs compared to patients, with physicians systematically underestimating overall QoL, social functioning, and role functioning. Interestingly, patients in the UK expected the orthotic device to foremost enable them to engage in ‘normal’ daily activities and taking part in social events and gatherings which represent relevant QoL domains.^1^ Hence, demands and potential benefits of MP-SSCOs in domains such as participation, mental well-being and QoL reported by HCPs in our study might be underestimated.

Yang et al. reported that patients with neurologic conditions are mostly interested in restoring both walking and standing, whereas patients with musculoskeletal injuries had a single focus on normal walking.^10^ Problems of orthosis use related to normal walking in patients with damage to the nervous system were mentioned mostly with regard to restricted mobility, skin injury due to excessive rubbing, orthosis durability and material-related issues. For those focusing on standing, material-related issues dominated. Problems in orthosis use by patients with musculoskeletal injuries were restricted mobility and material-related issues.^10^

The post-fall syndrome (depression, fear of falling and other psychological problems) is a common consequence of repeated falls, with up to 40% of patients not reporting recent falls and up to 70% of recent fallers reporting fear of falling.^19^ Loss of self-confidence as well as social withdrawal, confusion and loneliness can occur, even if there has been no injury. Reduced physical and functional activity is associated with fear and anxiety of falling - strong correlations have been found between fear and poor postural performance.^20^ Hence, the reported reduction in fall rate in our study indicates the potential for improved self-confidence and better QoL with a MP-SSCO.

Our results underline the need for proper alignment of orthosis components to reflect the individual patients’ anatomy. This is in line with Yang et al. as their results show that users with the same symptoms may use different orthoses.^10^ Our results confirm the need for incorporating patient relevant outcomes in real-life settings into high-quality research of orthotic devices for knee instability related to neuromuscular and central nervous system conditions.^3^

### Study limitations:

Despite having interviewed experts with different expertise and professional backgrounds, the low number of interviewees might contribute to potential bias. However, in Germany, there are only a few expert centres experienced both in treating and fitting patients with muscular knee instability and MP-SSCO fitting, resulting in an appropriate representation of centres’ experience.

Furthermore, the fact that results were obtained from a health care professionals’ point of view might have led to different perceptions compared to direct patient interviews or surveys. While formerly HCP-derived patient information was perceived to be superior to those collected from studied subjects,^21^ more recently patient self-reported outcomes are considered as an equally valuable contribution in the health sciences, where interviewer and physician assessments are understood to be complementary to self-assessed health measures.^22,23^ Furthermore, studies showed that results obtained from HCPs consistently underrate the positive impact on mental well-being and hence experts’ estimates might serve as conservative estimate of patients’ outcomes and needs.^18^

## CONCLUSION

Patients with muscular knee instability following neuromuscular or central nervous system injuries or conditions who use KAFOs/SCOs are suffering from restricted mobility, emotional strain and impaired gait patterns. Advanced orthotic technology might contribute to better QoL of patients, improved gait patterns with subsequent reduction of long-term consequences and perceived reliability of the orthosis. In terms of safety, a substantial decrease in the frequency of falls with MPSSCO compared to non-microprocessor-controlled KAFOs was reported. Advanced orthotic devices may enhance physical and psychological health and well-being by enabling patients to pursue their daily routines. In selected patients who are unable to be fitted with non-microprocessor-controlled KAFO/SCO, mobility might be regained through MP-SSCOs with the additional benefit of spending less time in a wheelchair or even discontinuing its use. Advanced orthoses require even more interdisciplinary rehabilitation with a standardized outcomes assessment comprising instruments for gait analysis and assessing the number of falls as well as individual participation in activities of daily living.

## DECLARATION OF CONFLICTING INTERESTS

Bernd Brüggenjürgen, Frank Braatz, Bernhard Greitemann, Axel Ruetz, Friedemann Steinfeldt, Daiwei Yao, and Christina Stukenborg-Colsman received lecture fees; Heiko Drewitz is an employee of Otto Bock; Michael Schäfer, Wolfgang Seifert have collaborations with the sponsor. Claudia Weichold none to be declared.

## AUTHOR CONTRIBUTION

**Bernd Brüggenjürgen**:

Conception and design, analysis and drafting of the paper, interpretation of the data; revising it critically for intellectual content and final approval of the version to be published.

**Frank Braatz, Bernhard Greitemann, Heiko Drewitz, Axel Ruetz, Michael Schäfer, Wolfgang Seifert, Friedemann Steinfeldt, Claudia Weichold, Daiwei Yao, Christina Stukenborg-Colsman**:

Interpretation of the data; revising it critically for intellectual content and final approval of the version to be published.

## SOURCES OF SUPPORT

This work was supported by a grant of Otto Bock Healthcare Products GmbH, Wien.

## ETHICAL APPROVAL

Expert participants were only selected, if willingness to devote time and interest to the specific topic was stated, as well as informed consent to participate was provided. As this survey was neither a notifiable clinical study according to §47 par. 3 MDPG nor an epidemiologic study with individual patient reference, an ethics committee vote did not apply.

## References

[1] O’Connor J, McCaughan D, McDaid C, Booth A, Fayter D, Rodriguez-Lopez R, et al. Orthotic management of instability of the knee related to neuromuscular and central nervous system disorders: systematic review, qualitative study, survey and costing analysis. Health technology assessment. 2016;20(55):1-262. DOI: 10.3310/hta20550 PMC4983707 27477023

[2] Fox JR, Lovegreen W. 22 - Lower Limb Orthoses. In: Webster JB, Murphy DP, editors. Atlas of Orthoses and Assistive Devices (Fifth Edition). Philadelphia: Elsevier; 2019; 239-46.e1.

[3] McDaid C, Fayter D, Booth A, O’Connor J, Rodriguez-Lopez R, McCaughan D, et al. Systematic review of the evidence on orthotic devices for the management of knee instability related to neuromuscular and central nervous system disorders. BMJ Open. 2017;7(9):e015927. DOI: 10.1136/bmjopen-2017-015927 PMC5588970 28877943

[4] Probsting E, Kannenberg A, Zacharias B. Safety and walking ability of KAFO users with the C-Brace((R)) Orthotronic Mobility System, a new microprocessor stance and swing control orthosis. Prosthet Orthot Int. 2017;41(1):65-77. DOI: 10.1177/0309364616637954 27151648 PMC5302081

[5] Arazpour M, Ahmadi F, Bani MA, Hutchins SW, Bahramizadeh M, Ghomshe FT, et al. Gait evaluation of new powered knee-ankle-foot orthosis in able-bodied persons: a pilot study. Prosthet Orthot Int. 2014;38(1):39-45. DOI: 10.1177/0309364613486917 23660383

[6] Tian F, Hefzy MS, Elahinia M. State of the art review of knee– ankle–foot orthoses. Ann Biomed Eng. 2015;43(2):427-41. DOI: 10.1007/s10439-014-1217-z 25631201

[7] Deems-Dluhy S, Hoppe-Ludwig S, Mummidisetty CK, Semik P, Heinemann AW, Jayaraman A. Microprocessor controlled knee ankle foot orthosis (KAFO) vs stance control vs locked KAFO: A randomized controlled trial. Arch Phys Med Rehabil. 2021;102(2):233-44. DOI: 10.1016/j.apmr.2020.08.013 32976844

[8] Schmalz T, Pröbsting E, Auberger R, Siewert G. A functional comparison of conventional knee–ankle–foot orthoses and a microprocessor-controlled leg orthosis system based on biomechanical parameters. Prosthet Orthot Int. 2016;40(2):277-86. DOI: 10.1177/0309364614546524 25249381

[9] Daines KJF, Farah J, Baddour N, Duke C, Bhatti J, Lemaire ED. Preliminary kinematic and kinetic evaluation of a modular microprocessor-controlled stance-control knee-ankle-foot orthosis. CMBES Proceedings. 2019;42(0).

[10] Yang BS, Chen YW, Tong JR. User experience of lower-limb orthosis. Assist Technol. 2018;30(5):267-73. DOI: 10.1080/10400435.2017.1322157 28598300

[11] Borsci S, Londei A, Federici S. The Bootstrap Discovery Behaviour (BDB): a new outlook on usability evaluation. Cogn Process. 2011;12(1):23-31. 21046191 10.1007/s10339-010-0376-6

[12] Ravneberg B. Usability and abandonment of assistive technology. J Assist Technol. 2012;6(4):259-69. DOI:10.1108/17549451211285753

[13] Söderström S, Ytterhus B. The use and non-use of assistive technologies from the world of information and communication technology by visually impaired young people: a walk on the tightrope of peer inclusion. Disabil Soc. 2010;25(3):303-15. DOI:10.1080/09687591003701215

[14] Klandermans B, Staggenborg S. Methods of Social Movement Research. Boston: University of Minnesota Press; 2002.

[15] Moretti F, van Vliet L, Bensing J, Deledda G, Mazzi M, Rimondini M, et al. A standardized approach to qualitative content analysis of focus group discussions from different countries. Patient Educ Couns. 2011;82(3):420-8. DOI: 10.1016/j.pec.2011.01.005 21292424

[16] Cho JY, Lee E-H. Reducing Confusion about Grounded Theory and Qualitative Content Analysis: Similarities and Differences. Qual Rep. 2014; 19. DOI:10.46743/2160-3715/2014.1028

[17] Santer J, MacDonald S, Rizzone K, Biehler S, Beiswenger T. Strategies for gait retraining in a collegiate runner with transfemoral amputation: A case report. Int J Sports Phys Ther. 2021;16(3):862-9. DOI: 10.26603/001c.23671 34123538 PMC8169020

[18] Wilson KA, Dowling AJ, Abdolell M, Tannock IF. Perception of quality of life by patients, partners and treating physicians. Qual Life Res. 2000;9(9):1041-52. DOI: 10.1023/a:1016647407161 11332225

[19] Tinetti ME, Mendes de Leon CF, Doucette JT, Baker DI. Fear of falling and fall-related efficacy in relationship to functioning among community-living elders. J Gerontol. 1994;49(3):M140-7. DOI: 10.1093/geronj/49.3.m140 8169336

[20] Maki BE, Holliday PJ, Topper AK. Fear of falling and postural performance in the elderly. J Gerontol. 1991;46(4):M123-31. DOI: 10.1093/geronj/46.4.m123 2071833

[21] Kriegsman DM, Penninx BW, van Eijk JT, Boeke AJ, Deeg DJ. Self-reports and general practitioner information on the presence of chronic diseases in community dwelling elderly. A study on the accuracy of patients’ self-reports and on determinants of inaccuracy. J Clin Epidemiol. 1996;49(12):1407-17. DOI: 10.1016/s0895-4356(96)00274-0 8970491

[22] Ferraro KF, Su YP. Physician-evaluated and self-reported morbidity for predicting disability. Am J Public Health. 2000; 90(1):103-8. DOI: 10.2105/ajph.90.1.103 10630145 PMC1446128

[23] Smith KV, Goldman N. Measuring health status: self-, interviewer, and physician reports of overall health. J Aging Health. 2011;23(2):242-66. DOI: 10.1177/0898264310383421 21041293 PMC3727648

